# Does delaying curative surgery for colorectal cancer influence long-term disease-free survival? A cohort study

**DOI:** 10.1007/s00423-021-02251-4

**Published:** 2021-07-11

**Authors:** Stephanie Garcia-Botello, J. Martín-Arevalo, C. Cozar-Lozano, A. Benitez-Riesco, D. Moro-Valdezate, V. Pla-Martí, A. Espí-Macías

**Affiliations:** 1 Colorectal Surgery Unit, Biomedical Research Institute INCLIVA, Hospital Clinico Universitario, Valencia, Spain; 2 Department of Surgery, Universidad de Valencia, Valencia, Spain

**Keywords:** Wait list time, Colorectal cancer, Survival

## Abstract

**Background:**

Surgical wait list time is a major problem in many health-care systems and its influence on survival is unclear. The aim of this study is to assess the impact of wait list time on long-term disease-free survival in patients scheduled for colorectal cancer resection.

**Materials and methods:**

A prospective study was carried out in patients with colorectal cancer scheduled for surgery at a tertiary care center. Wait list time was defined as the time from completion of diagnostic workup to definitive surgery and divided into 2-week intervals from 0 to 6 weeks. The outcome variables were 2-year and 5-year disease-free survival.

**Results:**

A total of 602 patients, 364 (60.5%) male, median age 73 years (range = 71) were defined. The median wait list time was 28 days (range = 99). Two and 5-year disease-free survival rates were 521 (86.5%) and 500 (83.1%) respectively. There were no differences in 2-year or 5-year disease-free survival for the whole cohort or by tumor stage between wait list time intervals except for AJCC stage II tumors which showed a higher 5-year disease-free survival for the 2–4 and 4–6-week wait list time interval (p = 0.021).

**Conclusions:**

Time from diagnosis to definitive surgery up to 6 weeks is not associated with a decrease in 2-year or 5-year disease-free survival (DFS) in AJCC stage I through III colorectal cancer patients. These are important findings in the light of the COVID-19 pandemic and offer a window of opportunity for preoperative optimization and prehabilitation.

## Introduction

Colorectal cancer is the most commonly diagnosed gastrointestinal cancer. In 2018, it represented 1.8 million cases and 881,000 deaths worldwide and was responsible for 1 in 10 cancer deaths [1]. It also represented 12.8% of all cancers in Europe with a 12.6% mortality rate [2]. Waiting lists for surgery and treatment for colorectal cancer are a problem in most countries, and there is conflicting evidence as to whether time from diagnosis to surgery has an influence on postoperative complications and long-term survival. Gort et al. reported that time to treatment and stage were independently associated with 3-year disease-free survival in rectal cancer patients [3]. Yun et al. reported similar results where treatment delays beyond 1 month were not associated with worse survival in a variety of gastrointestinal cancers including colon cancer, but did show a decrease in survival for rectal cancer [4]. Other authors have recently suggested a 3–6-week ideal time frame for first treatment of colorectal cancer patients, with an 8 to 14% higher 5-year survival rate for stage I–III colon cancer patients [5].

The global COVID-19 pandemic in 2020 has led to overloading and collapse of many national health systems with a need to defer treatment for many non-COVID-19 serious acute and chronic conditions [6, 7]. Several medical associations have published guidelines and practice parameters for the management and triage of these conditions during the COVID-19 crisis, offering alternative options during the lack of available hospital resources. Among these are those belonging to the Italian, Spanish, and the UK colorectal societies [8], published on their respective websites [9, 10]. Recommendations include triaging cancer patients according to the urgency of each case and the need for immediate treatment. These groups include immediate treatment for emergency cases at risk of obstruction or perforation, deferring earlier stage cases past the pandemic crisis or prolonging neoadjuvant therapies for more advanced cases.

The aim of this study was to assess the impact of the wait list time (WLT) on long-term overall disease-free survival in patients scheduled for colorectal cancer resection and assist WLT management during the COVID-19 pandemic or other future crisis.

## Materials and methods

A prospective cohort single-center study was carried out in patients undergoing definitive surgery for colorectal cancer from 2012 to 2017 and followed up until March 2020. Data was collected from a prospective institutional clinical database and subsequently analyzed. All patients were operated on by one of the 5 members of a highly specialized colorectal unit from a tertiary hospital and received standard multimodal enhanced recovery pre- and postoperative care without prehabilitation. The choice to perform laparoscopic or open surgery was up to surgeon preference and patient or tumor characteristics. Patients who had American Joint Committee on Cancer (AJCC) stage I through III confirmed adenocarcinomas were selected, with “colon” or “upper rectum” as their primary site of malignancy according to International Classification of Diseases for Oncology, Third Edition topography code. Patients were excluded if they had emergency surgery, more than one primary site, synchronous metastasis, or peritoneal carcinomatosis at the time of surgery, and if they received neoadjuvant chemoradiotherapy or a local transanal excision. AJCC stage was determined postoperatively by pathological staging in all cases.

WLT was defined as the time between diagnosis and definitive surgery. Diagnosis was defined as completion of diagnostic workup which included colonoscopy with biopsy and abdominal computed tomography scan for colon cancers, with the addition of rigid proctoscopy, pelvic magnetic resonance imaging scan, and endorectal ultrasound scan for rectal tumors. The whole “fast-track” oncological diagnostic circuit takes between 7 and 10 days. WLT was divided into 2-week intervals ranging from 0 to 6 weeks. Patients who were more than 6 weeks on the waiting list were an insufficient number to create subgroups beyond this time-point and were grouped into the same category as over 6 weeks. The outcome variables were 2-year and 5-year disease-free survival (DFS) and excluded patients who died in the immediate postoperative period (30 postoperative days). Screening for disease progression was performed with 6 monthly serum carcinoembryonic antigen (CEA) levels and chest/abdominal computed tomography scan performed after elevation of CEA levels or at yearly intervals if the latter were normal. Standard adjuvant chemotherapy was administered 6 weeks after surgery for AJCC stage III tumors and stage II tumors with pathological risk factors for local or systemic recurrence. Other variables analyzed were age, sex, Charlson comorbidity index score, tumor stage, resection margins, surgical approach, and postoperative complications. Institutional board approval was obtained, and patients signed informed consent. The work has been reported in line with the STROCSS criteria. [11]

### Statistical analysis

A descriptive analysis was performed, and quantitative variables were tested for normality with the Shapiro–Wilk test. Qualitative data were expressed as n (%) and quantitative data as median (interquartile range). The Kruskal–Wallis test was used for non-parametric data and the chi-squared test for quantitative variables. The DFS for each time interval was calculated using the Kaplan–Meier curves and log-rank test to assess for differences between curves. Cox regression was used to assess if WLT was prognostic for DFS. Statistical analysis was carried with the IBM® SPSS® Statistics Version 26 for MAC. p < 0.05 was considered statistically significant.

## Results

A total of 707 patients with AJCC stage I through III colorectal cancer who did not receive preoperative chemoradiotherapy were initially included. After applying the remaining exclusion criteria (synchronous colon/rectal tumors (24), local excisions (17), anal squamous cell carcinoma (10), appendix tumors (3), metachronous tumors (3), gastrointestinal stromal tumors (2)), a cohort of 648 patients was defined. Twenty-four patients were lost to follow-up, and there were 22 postoperative deaths. Finally, 602 patients were included for analysis; 364 (60.5%) males with a median (range) age of 73 years (71) were defined for the study. Median follow-up was 51.5 months (98). Median WLT was 28 days (99). Median body mass index (BMI) was 26 (32), preoperative hemoglobin 12.3 g/dL (8.2), preoperative albumin 4.1 g/dL (0.9), and Charlson Comorbidity Index 5.5 (13). A total of 471 (78.2%) of tumors were located in the colon and 131 (21.7%) in the rectum. Laparoscopy was performed in 454 (75.4%); median DFS was 43 months (range = 98). Two-year and 5-year DFS rates were 521 (86.5%) and 500 (83.1%) respectively. Table 1 shows adjusted patient and tumor data by WLT 2 weekly intervals. There were significantly lower hemoglobin and albumin levels in the 0–2-week group and a higher proportion of laparoscopic procedures in the 4–6 and over the 6-week groups. Other patient and tumor characteristics showed no significant differences between WLTs.

**Table 1:** Adjusted patient and tumor characteristics per wait list time

	Wait list time				
Characteristics	0–2 weeks	2–4 weeks	4–6 weeks	> 6 weeks	p
n	84 (14)	233 (38.7)	150 (24.9)	135 (22.4)	
Age*	75 (58)	73 (53)	70 (67)	72 (52)	0.069
Male^+^	49 (58.3)	140 (60.1)	97 (64.5)	78 (57.8)	0.639
CCI*	6 (11)	5 (10)	5 (10)	6 (11)	0.542
Body mass index*	27 (23.2)	27.3 (24,1)	27.8 (22)	28.1 (31.7)	0.078
Preoperative Hg g/dL*	11.1 (9.7)	12.2 (11.6)	12.7 (10.8)	12.7 (9.7)	< 0.001
Preoperative Alb g/dL*	4 (2.7)	4.1 (6.1)	4.2 (2)	4.1 (1,7)	0.005
Laparoscopy	54 (64.3)	166 (71.2)	118 (78.7)	116 (85.9)	0.001
Operating time (min)*	135 (256)	130 (345)	140 (284)	150 (300)	0.236
Location					
Colon*	66 (78.6)	191 (82)	109 (72.7)	105 (77.8)	0.198
Upper rectum*	18 (21.4)	42 (18)	41 (27.3)	30 (22.2)	
AJCC stage					
I	21 (25)	55 (24)	43 (28.7)	41 (30.4)	0.153
II	42 (50)	94 (40.3)	55 (36.7)	56 (41.5)	
III	21 (25)	83 (35.6)	52 (34.7)	38 (28.1)	
Adjuvant chemotherapy	35 (41.7)	82 (35.2)	57 (38)	45 (33.3)	0.600
AJCC stage^+^					
I	7 (4.3)				
II	61 (24.8)				n.a
III	143 (73.7)				
Clavien-Dindo score					
0	53 (63.1)	155 (66.5)	89 (59.3)	81 (60)	
I**–**II	21 (25)	52 (22.3)	39 (26)	38 (28.1)	0.783
> III	10 (11.9)	26 (11.2)	22 (14.7)	16 (11.9)	
2-year DFS	72 (85.7)	198 (85)	136 (90.7)	115 (85.2)	0.400
5-year DFS	69 (82.1)	192 (82.4)	129 (86)	110 (81.5)	0.733
Postoperative deaths	0 (0)	11 (4,9)	7 (4,7)	4 (3)	0.235
5-year CRD	8 (9.5)	17 (7.3)	12 (8)	15 (11.1)	0.628

Results are expressed as n (% per wait list time) unless otherwise stated; *DFS* disease−free survival, *AJCC* American Joint Committee on Cancer, *Hg* hemoglobin levels, *Alb* serum albumin levels, *CRD* cancer−related deaths, *CCI* Charlson Comorbidity Index, *n.a.* not applicable.

*Median (range).

+n (% per tumor stage).

**Fig. 1:**
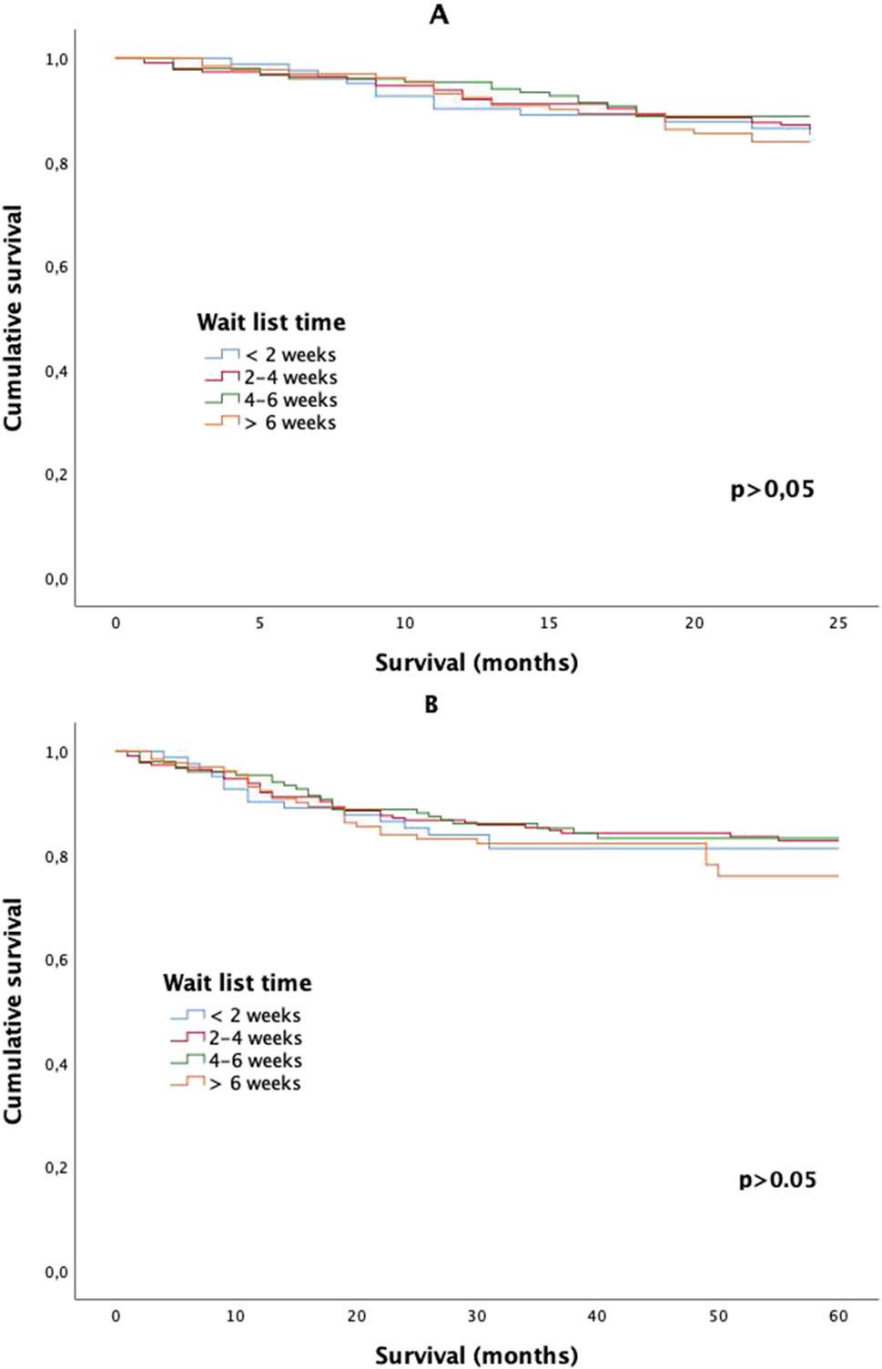
Cancer-specific survival curves per wait list time for the whole cohort. **A** Two-year disease-free survival. **B** Five-year disease-free survival

**Fig. 2:**
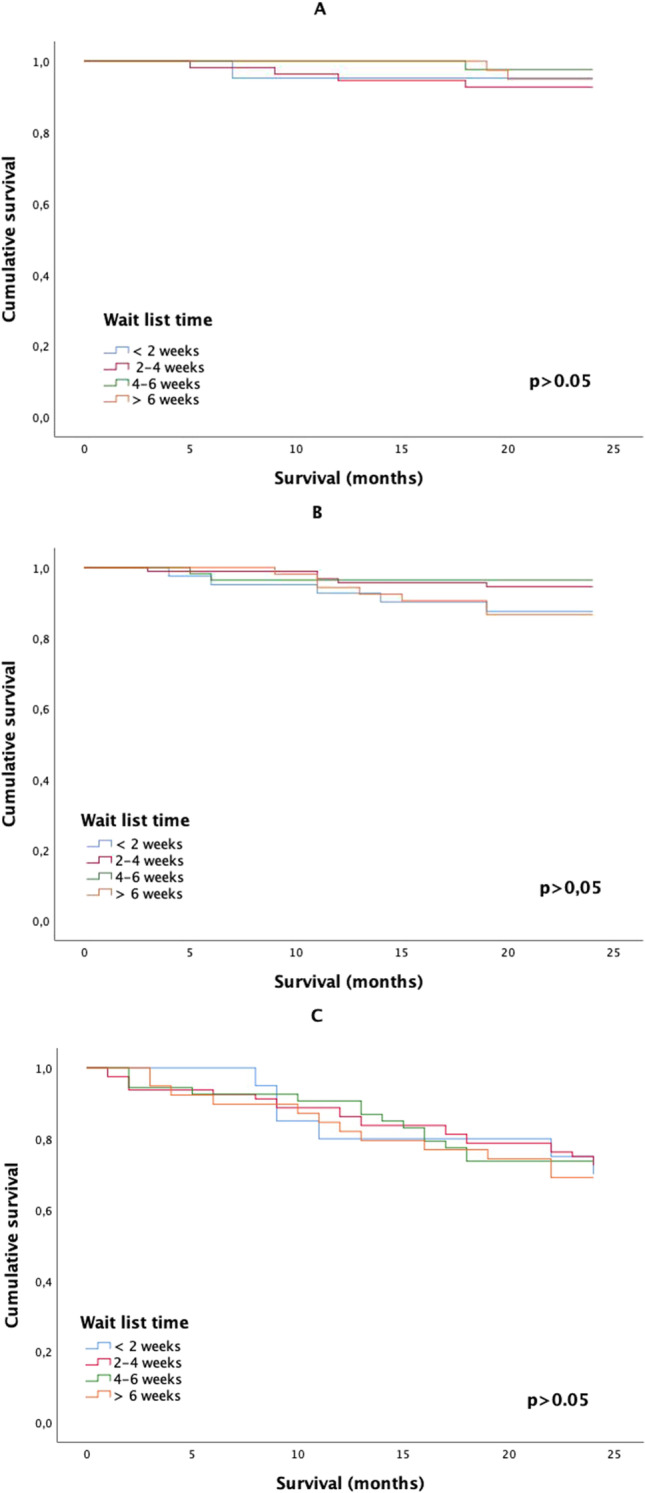
Cancer-specific 2-year disease-free survival curves of wait list time for patients scheduled for elective colorectal surgery (**A**) AJCC stage I disease, (**B**) AJCC stage II disease, and (**C**) AJCC stage III disease. AJCC American Joint Committee on Cancer

**Fig. 3:**
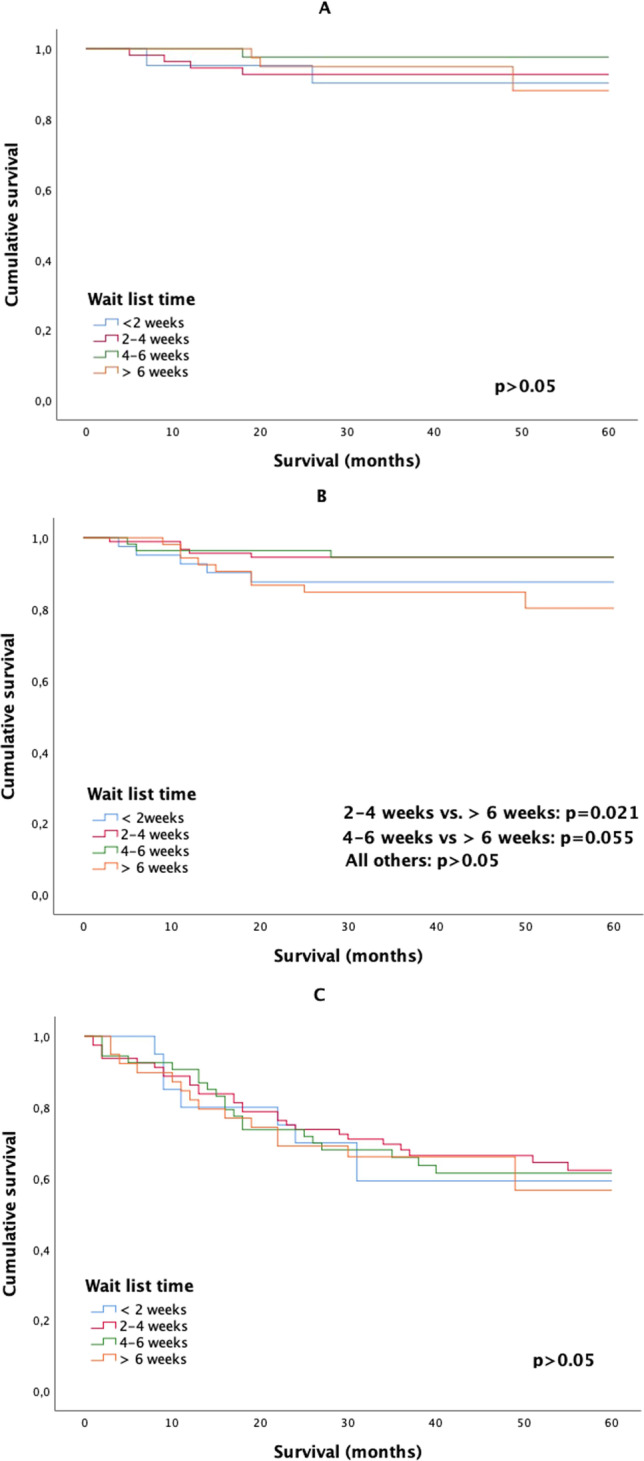
Cancer-specific 5-year disease-free survival curves of wait list time from for patients scheduled for elective colorectal surgery (**A**) AJCC stage I disease, (**B**) AJCC stage II disease, and (**C**) AJCC stage III disease [p > 0.05]. AJCC American Joint Committee on Cancer

Twenty-two patients (3.4%) died in the postoperative period. There were no significant differences between the WLT regarding postoperative deaths (p = 0.442). WLT did not statistically significantly impact on 2-year or 5-year DFS for the group as a whole (Fig. 1). The 2-year DFS showed no trend or differences between stages (Fig. 2). The 5-year DFS for the 2–4 and 4–6-week WLT interval was significantly higher for AJCC stage II tumors (Fig. 3). WLT was not found to be prognostic for DFS on Cox-regression analysis for the group either as a whole (p = 0.414) or when analyzed by tumor stage (p = 0.712).

## Discussion

This study analyses the possible effect of WLT on patients scheduled for AJCC stage I through III colorectal cancer who did not receive preoperative chemoradiotherapy. The time lapse between wait list inclusion and operation date was divided into 2-week intervals. The only sub-group of patients who showed a statistically significant difference between WLTs were patients with stage II AJCC cancers with longer 5-year DFS for the 2–4 and 4–6-week WLTs. In addition, there was a statistically significant increase in hemoglobin and albumin levels with WLT and a higher proportion of laparoscopic cases. There is a twofold interpretation to these results. Firstly, the 2–6-week WLT allows for adequate optimization of nutritional status and anemia, both factors having an impact on postoperative complications and long-term survival [12–15]. We did not have a formal trimodal prehabilitation program in place at the time of the study, though preoperative optimization of anemia, malnutrition, and comorbid conditions was carried out. Secondly, there may have been some selection bias, with priority given to patients with larger tumors, lower hemoglobin and albumin levels, and a possible risk of complications such as perforation or obstruction, making laparoscopy less likely in these cases, as reported by other authors [5, 16]. Surgery for colorectal cancer is necessary to obtain cure, and long WLTs cause considerable stress and worry for patients and relatives [17, 18] who usually push for prompt intervention. The results of this study reassure surgeons and patients that scheduling surgery between 2 and 6 weeks does not negatively affect DFS and allows for adequate optimization and preparation of the patient for the operation. There are several advantages to a 2–6-week WLT. It allows for availability of hospital resources and better preoperative oncological workup. In addition, a duration of 4 weeks is the recommended time to implement most prehabilitation programs improving nutritional, physical, and psychological patient status [19, 20]. Prehabilitation programs have been shown to improve postoperative patient recovery and decrease complications with the largest amount of literature in colorectal, thoracic, and urology patients [20–22]. Scheduling surgery between 2 and 6 weeks provides a window of opportunity to properly optimize and prehabilitate patients prior to surgery and improve surgical outcomes.

This WLT is particularly relevant at the moment. The COVID-19 pandemic has stretched and overloaded hospital capacity and resources worldwide. Medical and surgical beds have been occupied by COVID-19 patients. There has been a shortage in intensive care beds and operating rooms have been adapted and equipped for treating severely ill COVID-19 patients in many hospitals [7]. This has led to a severe shortage in available resources for surgical patients and most scheduled surgeries have been postponed. The few surgical resources available have been reserved for emergencies or very pressing oncological cases as recommended by the vast majority of surgical societies worldwide[6, 8–10, 23].

The effect of delaying surgery on cancer prognosis is unclear. This depends on tumor type, stage, and the length of time to surgery [3, 5]. Other factors known to influence survival are preoperative hemoglobin and albumin levels [15], location of the tumor [4], and preoperative comorbid conditions [3]. The vast majority of studies focus on the screening delays for colonoscopy and diagnosis of the cancer rather than on the surgical wait list from the date of inclusion to operation date [24, 25]. The impact of diagnostic delay on survival has been extensively studied with particular emphasis on colonoscopy screening programs [24, 26]. T∅rring et al. studied the results from 11,720 patients from five European data sets and found that longer diagnostic intervals in the primary care setting were associated with more advanced colorectal cancers. Conversely, specialist intervals up to 60 days from diagnosis were inversely proportional to cancer stage [27]. There are four time points in the run up to treatment where a delay may occur. Onset of symptoms is often preceded by a long asymptomatic phase with increasing degeneration [28]; so initially, there is the time that the patient takes for symptom recognition and consultation with the primary care physician [29]. Following this, there is the delay in endoscopic diagnosis followed by the hand-over of patient and results to the surgeon or oncologist, and finally the delay from diagnosis to treatment. The surgical WLT of 6 weeks may be only a drop in the ocean, and not be the major factor influencing long-term DFS when compared to the time from first symptom to diagnosis, which could be several months [24, 26, 30].

Recommended waiting times vary between countries with a clear example of a 2-week circuit in the UK which has not led to diagnosis of earlier stage cancers or improved long-term survival [31], or the socially acceptable rather than medically based standard published in the Netherlands [32]. The WLT may also depend on infrastructure of high-volume vs. low-volume hospitals where hospital crowding may play an important role [4]. Other authors also report on factors influencing WLT. Gort et al. [3] divide WLT in above or below 7 weeks and found that WLT below 7 weeks had a positive influence on survival for rectal cancer. Similar results were found by Yun et al. [4] who described a worse survival for rectal but not for colon cancer patients with WLTs over 1 month. Kucejko et al. [5] recently reported that a 3–4 week WLT was associated with the highest long-term survival for colon cancer and Turaga et al. [16] report that most cancer WLTs can be postponed more than 4 weeks from diagnosis without influencing long-term survival. Other authors have found no significant influence of WLTs on long-term survival [24, 25, 33, 34] albeit most studies focus on WLTs below 6 weeks. Our results are similar to those reported by Kaltenmeier et al., who found that wait list times greater than 30 days or within the first week independently increased mortality risk [35].

The limitations of this study are that it is a single-center retrospective analysis of data from a prospective institutional database. The lack of statistically significant differences in 5-year DFS for AJCC stage I and III cancers according to WLT could be attributed to the number of patients included in the study, albeit large for a single-center study, would probably require a multi-center trial or national database analysis to fully answer this question. This was a non-randomized study and the decision to operate at any particular time was based on order of wait list inclusion and on the possible risk of preoperative tumor-related complications. Ethical issues together with wait list campaigns and recommendations did not allow WLTs longer than 14 weeks, and the majority of cases were treated in the first 6 weeks. A minority of patients underwent surgery after this period, and the effect on WLT longer than 6 weeks remains unknown as there were too few patients in this group to create WLT subgroups beyond 6 weeks. There is currently no evidence for the maximum WLT. As previously mentioned, we did not assess the delay before referral for surgery. Time from diagnosis to treatment is only a part of the total delay. Patient socioeconomic level and access to health services has also been shown to influence survival and WLT and was not assessed in this study. The Spanish health system is public, and treatment is widely available to all patients regardless of their socioeconomic level, and therefore, this study did not address these issues. Nevertheless, the strengths of this study are the large number of patients included from a highly specialized colorectal unit with long follow-ups and rigorous database completion and statistics.

## Conclusions

In summary, time from diagnosis to definitive surgery up to 6 weeks is not associated with a decrease in 2-year or 5-year DFS in colorectal cancer patients. Early care is important to alleviate psychological stress in patients and families particularly when hospital resources are stretched as in the COVID-19 pandemic, but these results help alleviate some of these concerns. Furthermore, a 4-week window offers the possibility to optimize and prehabilitate the patient. Further well-designed prospective randomized studies with different WLT intervals could throw more light on these questions.

## Data Availability

Data is available on request.
